# Effectiveness of experiential learning with empowerment strategies and social support from grandmothers on breastfeeding among Thai adolescent mothers

**DOI:** 10.1186/s13006-017-0128-7

**Published:** 2017-08-22

**Authors:** Wilasinee Bootsri, Surasak Taneepanichskul

**Affiliations:** 1 Banmi Hospital, Lopburi, Thailand; 2 College of Public Health Sciences, Chulalongkorn University, Bangkok, Thailand

**Keywords:** Experiential learning, Empowerment, Social support, Grandmothers, Adolescent mothers

## Abstract

**Background:**

Grandmothers are important to successful breastfeeding because their knowledge, attitudes and experiences influence adolescent mothers’ decision to initiate and to continue breastfeeding. The purpose of this study was to assess the effectiveness of an experiential learning with empowerment strategies and social support (ELESSS) programme for grandmothers according to improvements in the rate and duration of exclusive breastfeeding (EBF); knowledge and attitude (KA) regarding breastfeeding; and perceived social support among adolescent mothers.

**Methods:**

A quasi-experimental study was conducted in two hospitals, Banmi as an intervention hospital and Inburi as a control hospital, between May 2015 and March 2016. Forty-two pairs of adolescent mothers and grandmothers were recruited from each hospital. At the baseline, grandmothers in the intervention group attended 2 days of an ELESSS programme, and they attended a refresher course 2 and 4 months after delivery. The grandmothers in the control group and adolescent mothers in both groups received the routine programme. Participants were assessed at the baseline and at two and 6 months after delivery to determine the rate and duration of EBF, KA regarding breastfeeding and perceived social support.

**Results:**

Adolescent mothers in the intervention group had the EBF rate at 6 months of around 29%, whereas the control group had the EBF rate at 6 months of about 5%, and the proportion of EBF in the intervention group was six times that of the control group. The median EBF duration in the intervention group was 90 days, while the control group was 0 day. A repeated measure ANOVA analysis showed that the intervention group’s participants had significantly better knowledge and attitudes towards breastfeeding, while the adolescent mothers in the intervention group had a significantly higher perceived level of social support.

**Conclusion:**

The ELESSS programme proved to be effective in increasing the rate and duration of EBF in adolescent mothers. Grandmothers are key to promoting, protecting and supporting breastfeeding.

**Trial registration:**

ClinicalTrials.in.th: TCTR20161001002

## Background

Breastfeeding is recognised as the gold standard for infant feeding and the best way to enhance the health of mothers and infants. The World Health Organization (WHO) and the United Nations Children’s Fund (UNICEF) recommend that all infants worldwide should breastfeed exclusively for the first 6 months of life, followed by partial breastfeeding combined with an appropriate diet until the age of 2 years or beyond [1]. Exclusive breastfeeding (EBF) is defined as “an infant’s consumption of human milk with no supplementation of any type (no water, no juice, no nonhuman milk, and no foods) except for vitamins, minerals, and medications until six months” [2]. Global Nutrition Targets 2025 increased the rate of exclusive breastfeeding in the first 6 months to at least 50% [3]. In 2012, a UNICEF report found that only around 12.3% of Thai mothers breastfed their babies exclusively for the first 6 months [4]. The national database does not record EBF rates among Thai adolescent mothers, but previous research has shown adolescent mothers have lower rates of breastfeeding than do adult mothers [5–7].

Adolescent mothers may choose not to breastfeed because they are generally unprepared for motherhood and experience changing emotions, making them impatient to satisfy the infant’s demands [8]. Several factors may explain the lower breastfeeding rates among adolescent mothers, such as a poor education and low income, returning to school, embarrassment about breastfeeding in public, lack of confidence in their ability to breastfeed and unease with the act of breastfeeding [5, 9–11].

Grandmothers represent influential persons in breastfeeding promotion. Their knowledge, attitudes and experiences influence mothers’ decisions to initiate and continue breastfeeding [12, 13]. In Thai society, family members pay respect to their elderly relatives and they obey their advice, including recommendations about breastfeeding [14].

There have been few experimental studies conducted specifically on grandmothers’ influence on adolescent mothers to promote EBF for the first 6 months after birth. Therefore, this study’s objective was to compare the effectiveness of ELESSS programme for grandmothers with adolescent mothers and the routine standard knowledge of breastfeeding techniques to determine the rate and duration of EBF, KA regarding breastfeeding and perceived social support in adolescent mothers and grandmothers.

## Methods

A quasi-experimental study was conducted in two hospitals located in Banmi, Lopburi Province and Inburi, Sing Buri Province, Thailand between May 2015 and March 2016. These hospitals were selected by purposive sampling according to their higher rates of adolescent pregnancy than the WHO standard (adolescent pregnancy should not exceed 10%), their lower EBF rates and their similar sociodemographic data. In addition, the hospitals share similar patient bed numbers, staff numbers and clinical guidelines for taking care of pregnant women. Both hospitals are considered general hospitals under the Ministry of Public Health, Thailand. The two hospitals were assigned randomly to the intervention (Banmi Hospital) and control (Inburi Hospital) groups. The sample size was calculated based on the proportions of outcome events in the two population groups [15].

The sample size was estimated for α = 5%, β = 20%, a 1:1 ratio between the intervention group and control group, a 5% prevalence of EBF at 6 months in the control group (Social Medicine Department, Inburi Hospital Report, 2013) and the expected outcome of 30% EBF at 6 months in the intervention group, as recommended in the 10th National Economic and Social Development Plan 2007–2011. Furthermore, with the addition of a 20% withdrawal rate, the total sample size in each group was 42 pairs of adolescent mothers and grandmothers, and the overall sample size was 168 participants (84 adolescent mothers and 84 grandmothers).

The inclusion criteria for the adolescent mothers included pregnant Thai women aged between 10 and 19 years who were pregnant for the first time or having their first baby and who had normal-looking breasts and nipples. Pregnant women who had serious diseases or a contraindication to breastfeeding were excluded. The grandmothers were between 35 and 60 years old, were living in the same house or nearby to the adolescent mother, had experience caring for infants and were willing to participate in this study. For the infants, the APGAR scores at the fifth minute were equal to seven points or higher and the birth weight ranged between 2500 and 4000 g. Infants who had problems with sucking, such as a cleft lip and cleft palate, severe tongue-tie and other contraindications to breastfeeding, were excluded.

### Training of interviewers

The interviewers were standardised by attending a 1 day training programme. Four nurse practitioners (two from each hospital) were trained with an interview questionnaire and a telephone call from the researcher. The same two nurses and four other nurses from Banmi attended a 3 day training programme to include education and teaching techniques taught by breastfeeding experts. Furthermore, the health volunteers attended a 1 day training programme specific for home visits and was conducted by the researcher.

### Intervention group

The ELESSS programme is based on the experiential learning, empowerment and social support theory [16–18]. At the baseline, grandmothers attended a 6 h, 2 day course and they learned about the benefits of breastfeeding, the composition and function of the breasts, the production and release of milk, latching on and positioning the baby, expressing the milk, storing the breast milk, feeding with a cup, solving breastfeeding problems and the grandmothers’ role in breastfeeding support. In the postpartum ward, the researcher and the nursing team provided grandmothers with breastfeeding knowledge and practice for 1 h to help them and the adolescent mothers care for their children. In the second and fourth months after delivery, the grandmothers of the adolescent mothers still breastfeeding their babies with only breast milk received an educational booster session, shared their problems and discussed breastfeeding; the researcher conducted the session for 1 h.

### Control group

The routine programme in the prenatal and postnatal clinic, was attended by the participants in the control group and the adolescent mothers in the intervention group, included breastfeeding and childcare education.

### Data collection

Participants in both groups received a face-to-face interview at the baseline, as well as at 2 months and 6 months after delivery. A telephone interview was conducted with the adolescent mothers from both groups about exclusive breastfeeding in the last 24 h at 7 days, 14 days, 1 , 2, 3, 4, 5 and 6 months after delivery.

### Questionnaires

The questionnaire included sociodemographic characteristics, gestational age at first visit to an antenatal care clinic, infant birth weight, experience with EBF and intention to have their grandchildren breastfeed among grandmothers, breastfeeding knowledge, attitudes towards breastfeeding, breastfeeding practice and perceived social support. Three experts in public health, including an expert in breastfeeding, an expert in maternity and infant care and an expert in research methodology, validated the questionnaire.

A pilot study was carried out to test the questionnaire’s reliability. The breastfeeding knowledge questionnaire included 20 items and the answers were “true” and “false.” The KR-20 values of the adolescent mothers and grandmothers’ knowledge questionnaires were 0.71 and 0.72, respectively. The attitude towards breastfeeding questionnaire included 15 items to determine the level of agreement with each question. A 5-point Likert scale with categories ranging from strongly agree to strongly disagree was used to document the responses to all questions. The Cronbach’s Coefficient Alpha value for the adolescent mothers and grandmothers was 0.72 for both. The perceived social support questionnaire was applied to assess the support provided by grandmothers. The scale consisted of 12 questions, and a 5-point rating scale ranged from strongly agree to strongly disagree. The Cronbach’s Coefficient Alpha was 0.87.

### Statistical analysis

Descriptive statistics, a chi - square test, Fisher’s exact test, the Mann–Whitney *U* test and a t-test were used to compare the differences between the intervention and the control groups at the baseline. A chi-square test was used to explore the effect of the intervention on the EBF rate. The Mann–Whitney *U* test was used to compare the differences in breastfeeding duration. A t-test was used to compare the differences between the intervention and the control groups to determine perceived social support at 6 months after delivery. A repeated measure ANOVA was also used to compare changes in outcome across time in knowledge and attitudes towards breastfeeding. The time-by-group interaction effect assessed the differences in outcome change. A post-hoc test (Bonferroni) was used to assess differences in outcome measures between the groups. The data were analysed with SPSS package version 16.0. All analyses used a 95% confidence interval (CI) and a statistically significant *p*-value of less than 0.05.

## Results

### Characteristics of sample

Among adolescent mothers, there were no statistically significant differences in age, parity, education, marital status, occupation, income, gestational age at first visit to an antenatal care clinic, mode of delivery and infant birth weight between the intervention and control groups (Table 1). Among grandmothers, there were no statistically significant differences in age, relationship with the adolescent, education, occupation, experience with EBF and the intention to breastfeed the infant between the intervention and control groups (Table 2).

**Table 1:** Baseline characteristics of adolescent mothers (*n* = 84)

Characteristics	Intervention group (*n* = 42)	Control group (*n* = 42)	*p* - value
Age (years)			0.791 (a)
Mean ± SD	17.88 ± 1.19	17.81 ± 1.27	
Min - Max	15–19	15–19	
Parity, (*n*, %)			0.242 (b)
Primiparous	37 (88.1)	33 (78.57)	
Multiparous	5 (11.9)	9 (21.43)	
Education, (*n*, %)			0.873 (b)
Primary school	10 (23.8)	10 (23.8)	
Junior high school	17 (40.5)	20 (47.6)	
Senior high school or equivalent	9 (21.4)	8 (19)	
Higher than senior high school	6 (14.3)	4 (9.5)	
Marital status, (*n,* %)			1.00 (c)
Married	39 (92.86)	38 (90.48)	
Separated temporary	0 (0)	1 (2.38)	
Permanent separation	2 (4.76)	2 (4.76)	
Solitary	1 (2.38)	1 (2.38)	
Occupation, (*n*, %)			0.449 (c)
Student	7 (16.67)	3 (7.14)	
Employed	3 (7.14)	4 (9.52)	
Unemployed	32 (76.19)	34 (80.95)	
Business owner	0 (0.00)	1 (2.38)	
Income, (*n,* %)			0.470 (c)
No income	39 (92.86)	37 (88.10)	
< 5000	1 (2.38)	0 (0.00)	
5000–9999	1 (2.38)	3 (7.14)	
10,000–15,000	1 (2.38)	1 (2.38)	
> 15,000	0 (0.00)	1 (2.38)	
First ANC (weeks)			0.209 (d)
Median (IQR)	16.5 (9)	14 (9)	
Min - Max	6–30	5–29	
Mode of delivery, (*n*, %)			0.662 (b)
Normal labor	19 (45.24)	21 (50)	
Cesarean section	23 (54.76)	21 (50)	
Infant birth weight			0.704 (a)
Mean ± SD	3054.76 ± 313.221	3079 ± 274.087	
Min - max	2525–3635	2555–3610	

Significant at *p* - value <0.05, (a) = t-test, (b) = Chi-square, (c) = Fisher’s Exact test, (d) = Mann–Whitney *U* test

**Table 2:** Baseline characteristics of grandmothers (*n* = 84)

Characteristics	Intervention group (*n* = 42)	Control group (*n* = 42)	*p* - value
Age (years)			0.653 (a)
Mean ± SD	50.24 ± 6.72	49.55 ± 7.27	
Min - max	35–60	35–60	
Relation with adolescent mother, (*n*, %)			0.446 (b)
Mother	24 (57.14)	19 (45.24)	
Husband’s mother	8 (19.05)	15 (35.71)	
Grandmother	6 (14.29)	4 (9.52)	
Husband’s grandmother	2 (4.76)	1 (2.38)	
Aunt	2 (4.76)	3 (7.14)	
Education, (*n*, %)			0.673 (b)
Illiteracy	4 (9.50)	2 (4.80)	
Primary school	32 (76.20)	33 (78.60)	
Secondary school	5 (11.90)	7 (16.70)	
Higher than secondary school	1 (2.40)	0 (0)	
Occupation, (*n*, %)			0.055 (b)
Business owner	3 (7.14)	1 (2.38)	
Trade	3 (7.14)	3 (7.14)	
Employed	17 (40.48)	25 (59.52)	
Housewife	4 (9.52)	8 (19.05)	
Agriculturist	15 (35.71)	5 (11.90)	
Experience for exclusive breastfeeding, (*n*, %)			0.36 (b)
Ever	1 (2.38)	4 (9.52)	
Never	41 (97.62)	38 (90.48)	
Intention to feed grandchild, (*n*, %)			0.812 (b)
Only breast milk	4 (9.52)	8 (19.05)	
Breast milk + water	19 (45.24)	18 (42.86)	
Breast milk + water + formula milk	5 (11.90)	4 (9.52)	
Breast milk + water + complementary food	8 (19.05)	7 (16.67)	
Breast milk + water + complementary food + formula milk	6 (14.29)	5 (11.90)	

Significant at *p* - value <0.05, (a) = t-test, (b) = Fisher’s Exact test

### Rate, proportion, duration of exclusive breastfeeding

There was a statistically significant difference in the EBF and no EBF rates between the intervention and control groups at 6 months after delivery (*p* = 0.003). The proportion of EBF in the intervention group was approximately six times that of the control group. There was a statistically significant difference in EBF duration between the intervention and control groups (*p* < 0.001) (Table 3).

**Table 3:** Rate and duration of exclusive breastfeeding (*n* = 84)

Duration	Intervention group (*n* = 42)	Control group (*n* = 42)	*p* - value
EBF rate at 6 month n (%)			0.003 (a)
EBF	12 (28.6)	2 (4.8)	
NO EBF	30 (71.40)	40 (95.20)	
Duration of EBF (days)			
Median (IQR)	90 (30–180)	0 (0–0)	< 0.001 (b)
Min-Max	0–180	0–180	

Significant at *p* - value <0.05, (a) = Chi-square test, (b) = Mann–Whitney *U* test

### Breastfeeding knowledge

For both the adolescent mothers and grandmothers, there were no statistically significant mean difference in breastfeeding knowledge at the baseline between the intervention and the control groups; however, there were statistically significant differences at 2 and 6 months after delivery (*p* < 0.001 and *p* < 0.001, respectively) (Table 5).

Among the adolescent mothers, there were statistically significant differences in overall knowledge between the intervention and control groups (F = 4.172, *p* < 0.001), within measurements (F = 130.536, *p* < 0.001) and within the interaction effect between measurements depending on the group (F = 114.032, *p* < 0.001) (Table 4).

**Table 4:** Repeated measure ANOVA of knowledge and attitude toward breastfeeding (scores) in adolescent mothers and grandmothers (*n* = 168)

Source of variation	SS	df	MS	F	*p* - value
Adolescents					
Knowledge					
Between subjects					
Intervention	51,858.036	1	51,858.036	4.172	< 0.001
Error	1019.151	82	12.429		
Within subjects					
Time	700.310	1.309	534.888	130.536	< 0.001
Intervention x time	611.770	1.309	467.262	114.032	< 0.001
Error	439.921	107.360	4.098		
Attitude					
Between subjects					
Intervention	756,152.444	1	756,152.444	9.735	<0.001
Error	6369.222	82	77.673		
Within subjects					
Time	825.294	1.779	463.851	61.612	<0.001
Intervention x time	719.643	1.779	404.471	53.724	<0.001
Error	1098.397	145.896	7.529		
Grandmothers					
Knowledge					
Between subjects					
Intervention	50,858.730	1	50,858.730	2.052	<0.001
Error	2032.794	82	24.790		
Within subjects					
Time	387.175	1.575	245.798	40.098	<0.001
Intervention x time	496.381	1.575	315.128	51.407	<0.001
Error	791.778	129.164	6.130		
Attitude					
Between subjects					
Intervention	734,724.004	1	734,724.004	7.262	< 0.001
Error	8296.325	82	101.175		
Within subjects					
Time	616.056	2	308.028	40.827	< 0.001
Intervention x time	487.960	2	243.980	32.338	< 0.001
Error	1237.317	164	7.545		

*SS* sum of squares, *df* degree of freedom, *MS* mean squares, *F* F-test

For the grandmothers, there were statistically significant differences in overall knowledge between the intervention and control groups (F = 2.052, *p* < 0.001), within measurements (F = 40.098, *p* < 0.001) and within the interaction effect between measurements depending on the group (F = 51.407, *p* < 0.001) (Table 4).

The mean difference in breastfeeding knowledge between the intervention and control groups was the highest at 6 months (5.79 and 4.95 in adolescent mothers and grandmothers, respectively) (Table 5).

**Table 5:** Pairwise comparisons of the different measurements of knowledge and attitude toward breastfeeding (scores) in adolescent mothers and grandmothers (*n* = 168)

Variables	Intervention group	Control group	Mean difference	*p* - value
Adolescents				
Knowledge				
Baseline	11.43 ± 3.65	12.57 ± 2.66	1.14 ± 0.70	0.105
2nd month	17.86 ± 1.89	12.76 ± 2.11	5.10 ± 0.44*	< 0.001
6th month	18.62 ± 1.56	12.83 ± 2.16	5.79 ± 0.41*	< 0.001
Attitude				
Baseline	51.69 ± 5.736	52.79 ± 5.795	1.10 ± 1.26	0.387
2nd month	58.62 ± 5.66	52.93 ± 5.50	5.69 ± 1.22*	< 0.001
6th month	59.52 ± 5.14	53.12 ± 5.19	6.41 ± 1.13*	< 0.001
Grandmothers				
Knowledge				
Baseline	11.81 ± 3.94	13.33 ± 3.59	1.52 ± 0.82	0.068
2nd month	16.33 ± 3.46	12.62 ± 3.58	3.71 ± 0.77*	< 0.001
6th month	18.05 ± 2.26	13.10 ± 3.25	4.95 ± 0.61*	< 0.001
Attitude				
Baseline	52.50 ± 5.11	51.07 ± 6.95	1.43 ± 1.33	0.287
2nd month	58.88 ± 6.39	51.21 ± 6.69	1.67 ± 1.43*	< 0.001
6th month	58.62 ± 5.84	51.69 ± 6.19	6.93 ± 1.31*	< 0.001

Based on estimated marginal means

*The mean difference is significant at the 0.005 level

### Attitude toward breastfeeding

At the baseline (42 pairs of adolescent mothers and grandmothers), there were no statistically significant differences in attitudes towards breastfeeding between the intervention and control groups (*p* = 0.387 and 0.287, respectively); however, there were statistically significant differences at two and 6 months after delivery (*p* < 0.001 and *p* < 0.001, respectively) (Table 5).

Among the adolescent mothers and grandmothers (42 pairs of adolescent mothers and grandmothers in each group), there were statistically significant differences in overall attitudes between the intervention and control groups (*p* < 0.001), within measurements (*p* < 0.001) and within the interaction effect between measurements depending on the group (*p* < 0.001) (Table 4). The mean difference in attitudes towards breastfeeding between the intervention and control groups was highest at 6 months (6.41 and 6.93 in adolescent mothers and grandmothers, respectively).

### Perceived social support

Perceived social support was measured at 6 months after delivery for adolescent mothers’ perception of the supported provided by the grandmothers.

The average scores of the perceived social support of adolescent mothers in the intervention and control groups (Mean ± SD) were 55.36 ± 5.13 and 43.74 ± 5.54, respectively. There were statistically significant differences in the average scores of perceived social support at 6 months after delivery between the intervention and control groups (*t* = 9.964, *p* < 0.001) (Table 6).

**Table 6:** Comparison scores of perceived social support of adolescent mothers between the intervention and the control groups (*n* = 84)

Variable	Intervention (*n* = 42)	Control (*n* = 42)	t	*p* - value
Perceived social support (scores)			9.964	< 0.001*
Mean (SD)	55.36 (5.13)	43.74 (5.54)		
Min - Max	41–60	27–55		

*Significant at *p* - value <0.05, using independent t-test

## Discussion

This study showed that the ELESSS programme for grandmothers improved the rate; duration; and the knowledge and attitude (KA) regarding breastfeeding and perceived social support among adolescent mothers, and the improvement was sustained for 6 months. The present study indicated that the rate and duration of EBF in adolescent mothers whose grandmothers participated in the ELESSS programme were higher and longer than adolescent mothers whose grandmothers did not participate in this programme. It could be explained that during the group session, the grandmothers had the opportunity to provide information and to ask questions to provide a better understanding. Members have opportunities to express opinions and feelings while exchanging experiences with one another, receiving support or opposition from the group and gathering accurate advice from the researcher, who served as the group moderator. Thus, the sample group’s participants were made aware that some of their thoughts or beliefs were incorrect and that they had connected what they had learned with existing experiences.

Furthermore, the researcher provided them with new breastfeeding knowledge and breastfeeding policies to promote EBF, as most grandmothers had their children in the 1970s, 1980s and 1990s, a time when few Thai women engaged in EBF. In the control group, the significantly low rate of breastfeeding could be explained by the Thai culture, which, along with many countries around the world, believes water is necessary. Some of the most common reasons given are; it is necessary for life, quenches thirst, prevents and treats cold and constipation, soothes fretfulness and decreases jaundice and hiccup infants. Proverbs passed down from generation to generation advise mothers to give their babies water. Thai grandmothers think it is impossible through day do not drink water. Thai grandmothers did not know the exact meaning of ‘exclusive breastfeeding’ , as they thought EBF meant feeding infants with breast milk without formula, complementary except water [19, 20].

The study from Grassley and Nelms found that grandmothers who had not breastfed or who had limited knowledge of breastfeeding approached their daughters’ decisions to breastfeed in one of two ways [21]. One group of grandmothers did not oppose breastfeeding even though they were unable to offer practical support, whereas the other group was described by their daughters as unsupportive because they either tried to dissuade them from breastfeeding or quickly recommended feeding formula milk when breastfeeding was not going well. The present study is similar to a previous research paper that found counselling sessions for adolescent mothers and grandmothers increased the duration of EBF more in the intervention group than in the control group [22].

Ratisunthorn et al. revealed the effects of empowerment on adolescent mothers after implementing the programme, and the EBF duration in the intervention group was statistically longer than that in the control group (*p* < 0.01) [23]. This finding is inconsistent with that of Meglio et al. who conducted a randomised controlled trial project, Telephone Peer Support, for adolescent mothers; and they found ‘any breastfeeding’ duration did not differ significantly between the groups [24]. Furthermore, Kang et al. investigated the programme using education based on Freire’s empowerment education model, and the results revealed that the programme resulted in a significant improvement in the EBF rate in the intervention group compared to the control group [25].

A repeated measure ANOVA analysis in the present study showed that knowledge of and attitudes towards breastfeeding increased after the intervention. These results showed the effectiveness of the ELESSS programme to increase and maintain knowledge and attitudes in grandmothers for the first 6 months. This is consistent with a previous study that found that health promotion increased the knowledge of EBF in the intervention group, while the control group showed no difference [26]. Grassley et al. conducted the project ‘A Grandmother’s Tea’ , and the results showed knowledge scores in the intervention group were higher than in the control group, but there was no statistically significant difference between the two groups regarding attitudes towards breastfeeding [27]. The present study found participants in the intervention group had a higher score for breastfeeding practice than did those in the control group. This is consistent with a previous research paper that studied the impact of health education on breastfeeding. The results found that a health education programme produced a significant improvement in knowledge, attitudes and practice (KAP) regarding breastfeeding [28]. Furthermore, Handayani et al. indicated that mothers who had high KAP regarding breastfeeding exclusively breastfed their babies. Knowledge has a strong effect on breastfeeding practice (*p* < 0.001) and attitude has a weak influence on breastfeeding practice (*p* = 0.02) [29].

The results of the present study show adolescent mothers in the intervention group received higher social support from grandmothers than did those in the control group. It can be explained that the grandmothers who participated in the ELESSS programme had better skills and knowledge to support the adolescent mothers’ breastfeeding. During the group meeting, the researcher emphasised the importance of breastfeeding and the methods in which the grandmothers could assist the breastfeeding adolescent mothers based on social support. This is consistent with a previous study that found support from husbands, grandmothers and nurses encouraged mothers to engaged in EBF and continue the practice for a longer duration. The major reason for weaning breastfeeding was incorrect advice from grandmothers. However, instrumental support from grandmothers was predictive of EBF duration [30]. Furthermore, Srisawat et al. conducted research on the effect of grandmother support, and the results showed that first-time mothers perceived better social support from grandmothers in the intervention group than in the control group, and the mothers in the intervention group were satisfied with their grandmothers’ participation in the study [31].

The strength of this study was that it conducted home visits after the mothers were discharged from the hospital to assess breastfeeding practice, as telephone follow-ups may not produce accurate data; home visits enable the researcher to observe the real situation and the actual practice of infant care. A high response rate (100%) was also one of the study’s strengths. A limitation of the present study was a lack of random assignment due to the quasi-experimental design. Therefore, selection bias may be present. It may not be possible to generalise the findings to other populations, and it was not representative of all grandmothers and adolescent mothers. The Hawthorne effect [32] may have occurred in this study, because the intervention group knew they were being observed. Thai grandmothers may feel obligated to cooperate with the researcher so they ensure a good outcome. However, the ELESSS programme demonstrated effectiveness and acceptability that could be adapted into routine work by staff in the hospital and that could be implemented in other hospitals.

Future studies need to incorporate a longer follow-up, as WHO and UNICEF recommendations state that mothers should breastfeed exclusively for the first 6 months and then provide partial breastfeeding combined with an appropriate diet until the age of 2 years or beyond to establish an understanding of the effects and sustainability of and adherence to the intervention over time. Moreover, husbands, grandfathers and other male relatives should be incorporated into the programme to promote EBF in adolescent mothers.

## Conclusion

The results of the present study showed the effectiveness of ELESSS in improving the rate and duration of exclusive breastfeeding; knowledge, attitudes and practices regarding breastfeeding; and perceived social support; and the improvement was sustained for 6 months. Grandmothers are key to promoting, protecting and supporting breastfeeding if they show concern and understand new breastfeeding knowledge. Moreover, health care teams should support adolescent mothers and families in encouraging breastfeeding.

## Abbreviations

EBF: Exclusive breastfeeding
ELESSS: Experiential learning with empowerment strategies and social support
KAP: Knowledge, attitudes and practices
UNICEF: United Nations Children’s Fund
WHO: World Health Organization
